# Cognitive control in adults with high-functioning autism spectrum disorder: a study with event-related potentials

**DOI:** 10.3389/fpsyt.2023.1180827

**Published:** 2023-08-04

**Authors:** Laura Möde, Anna Borgolte, Erfan Ghaneirad, Mandy Roy, Christopher Sinke, Gregor R. Szycik, Stefan Bleich, Daniel Wiswede

**Affiliations:** ^1^Department of Psychiatry, Social Psychiatry and Psychotherapy, Hannover Medical School, Hannover, Germany; ^2^Asklepios, Psychiatric Hospital Ochsenzoll, Hamburg, Germany; ^3^Center of Systems Neuroscience, Hannover, Germany; ^4^Department of Neurology, University of Lübeck, Lübeck, Germany

**Keywords:** high-functioning, autism, cognitive control, flanker task, conflict adaptation, post-error slowing, ERN, Pe

## Abstract

**Introduction:**

Little is known about cognitive control in adults with high-functioning forms of autism spectrum disorder because previous research focused on children and adolescents. Cognitive control is crucial to monitor and readjust behavior after errors to select contextually appropriate reactions. The congruency effect and conflict adaptation are measures of cognitive control. Post-error slowing, error-related negativity and error positivity provide insight into behavioral and electrophysiological correlates of error processing. In children and adolescent with autism spectrum disorder deficits in cognitive control and error processing have been shown by changes in post-error slowing, error-related negativity and error positivity in the flanker task.

**Methods:**

We performed a modified Eriksen flanker task in 17 adults with high-functioning autism spectrum disorder and 17 healthy controls. As behavioral measures of cognitive control and error processing, we included reaction times and error rates to calculate congruency effects, conflict adaptation, and post-error slowing. Event-related potentials namely error-related negativity and error positivity were measured to assess error-related brain activity.

**Results:**

Both groups of participants showed the expected congruency effects demonstrated by faster and more accurate responses in congruent compared to incongruent trials. Healthy controls exhibited conflict adaptation as they obtained performance benefits after incongruent trials whereas patients with autism spectrum disorder did not. The expected slowing in reaction times after errors was observed in both groups of participants. Individuals with autism spectrum disorder demonstrated enhanced electrophysiological error-processing compared to healthy controls indicated by increased error-related negativity and error positivity difference amplitudes.

**Discussion:**

Our findings show that adults with high-functioning autism spectrum disorder do not show the expected upregulation of cognitive control in response to conflicts. This finding implies that previous experiences may have a reduced influence on current behavior in these patients which possibly contributes to less flexible behavior. Nevertheless, we observed intact behavioral reactions after errors indicating that adults with high-functioning autism spectrum disorder can flexibly adjust behavior in response to changed environmental demands when necessary. The enhancement of electrophysiological error-processing indicates that adults with high-functioning autism spectrum disorder demonstrate an extraordinary reactivity toward errors reflecting increased performance monitoring in this subpopulation of autism spectrum disorder patients.

## 1. Introduction

According to the DSM-V and ICD-11 diagnostic classifications, autism spectrum disorder (ASD) is a persistent neurodevelopmental condition that is categorized in different severity levels according to symptom intensity and the need for support. ASD is characterized by deficits in social communication including difficulties in social–emotional reciprocity, non-verbal communication, and deficits in developing, maintaining, and understanding relationships. Another core symptom of ASD are restrictive, repetitive patterns of behavior that often manifest in the form of stereotyped motor movements, insistence on sameness, inflexible adherence to routines or ritualized patterns of verbal and non-verbal behavior, that, for example, manifest as extreme distress in response to small changes in the environment (1, 2).

The pathogenesis of ASD is most presumably multifactorial with a strong genetic component that interacts with environmental risk factors, somatic, and psychiatric disorders as well as neurobiological and biochemical alterations (3, 4). Environmental risk factors comprise prenatal and perinatal factors as well as lifestyle aspects. Advanced maternal and paternal age have been linked to a higher rate of *de novo* mutations and epigenetic alternation that contribute to the development of ASD (5, 6). Maternal psychiatric disorders and maternal autoimmune diseases also heighten the probability of developing ASD (7). ASD has a strong genetic component with recent research suggesting that 74–93% of ASD risk is heritable (8) and having identified genetic variants that contribute to the probability of occurrence of ASD (3). In addition, ASD is associated with differences in brain development that lead to under-connectivity in large parts of brain areas and over- connectivity in frontal and occipital areas (3, 9). Recent studies suggest that the heterogeneous manifestation of ASD is partially due to individual reorganization of brain structures that vary interindividually depending on the person’s sensitivity toward environmental stimuli (10).

Given the heterogeneity of symptom manifestation ins ASD (1, 2), recent research has suggested that ASD can probably not be explained by a single neurobiological or cognitive approach. To deepen the understanding of the heterogenous symptom manifestation in ASD, cognitive characteristics of individuals with ASD provide a promising approach (11, 12). Ozonoff et al. (13) were the first to propose a causal link between executive dysfunction and ASD. Executive function (EF) is an umbrella term for a group of cognitive control processes that include shifting between mental sets or tasks, updating and monitoring of working memory contents, and inhibition of responses as well as planning abilities (14). A recent meta-analysis concluded that there is an overall effect of executive dysfunction in individuals with ASD across all domains (15) whereas other authors report stronger deficits in certain domains of EF while other domains remain relatively unimpaired (16). Several authors have proposed a causal link between executive dysfunction and behavioral rigidity, insistence on sameness, and problems with switching between tasks in ASD (17, 18). Nevertheless, a recent review concluded that research on executive dysfunction in ASD remains largely inconsistent (19).

In the present study, we focused on the ability to flexibly adapt behavior in response to a changing environment which is a crucial aspect of executive function and requires the capability to monitor thoughts and actions consistently and accurately. Thereby, enabling the selection of contextually appropriate responses while simultaneously inhibiting inappropriate responses (20). Whenever task-irrelevant and task-relevant information compete for attentional resources, cognitive control is essential to adjust behavior according to internal goals (21). The Conflict Monitoring Theory (CMT) argues that this is achieved by means of a cognitive system that monitors for conflicts, forwards corresponding information to a control processing center, and thereby initiates compensatory adjustments in control (14). Previous studies suggest that individuals with ASD demonstrate difficulties in monitoring one’s actions (22, 23) and exhibit alterations in response inhibition (24) resulting in difficulties to flexibly adjust cognitive processes in order to disengage form a task or situation and adapt behavior to new requirements (25). Moreover, the findings of Poljac et al. (26) indicate that individuals with ASD show higher task-switching costs and proposed that intentional control mechanisms and associated actions may contribute to behavioral rigidity in ASD. The described deficits in performance monitoring have been linked to a variety of ASD symptoms such as more pronounced restricted and repetitive behavior (26, 27), difficulties in social–emotional and social-cognitive areas, and empathy-related problems (28–30).

Cognitive control and conflict adaptation are examined using cognitive paradigms, such as the Eriksen flanker task (31). The flanker task allows to investigate routine behavior as well as behavioral adjustments provoking deviations from routines (e.g., in response to high conflict or errors). It has a low level of difficulty. Healthy adults usually commit less than 20 percent of errors. The flanker task allows participants to exhibit long sequences of correct responses, thereby enabling “routine behavior.” Additionally, participants respond more slowly and less accurately in incongruent trials (that comprise task-irrelevant and task-relevant information) because irrelevant stimuli also occupy cognitive resources. This effect, which is referred to as the congruency effect (21), has been reported in healthy controls (HC) as well as individuals with ASD (22, 28, 30, 32).

When successfully performing routine behavior, the congruency effect is less pronounced when the prior trial contained incongruent stimuli relative to a prior trial containing congruent stimuli. This is referred to as the Gratton effect (33). One of the most influential theories aiming to explain the Gratton effect is the CMT. It proposes that the Gratton effect is explicable by conflict adaptation arguing that high conflict in the previous trial (prime trial) induces top-down processes, thereby provoking increased cognitive control and enabling better performance in the current trial (probe trial). In the following, the trial of interest is indicated by an upper letter. The CMT suggests that reaction times (RT) are faster when incongruent trials follow incongruent trials (iI) than when incongruent trials follow congruent trials (cI) as a consequence of conflict adaptation (21). Alternatively, the Theory of Event Coding (TEC) (34) proposes that stimulus and response features are combined in an episodic memory representation, a so-called event file, influencing subsequent performance by retrieving the event file when one of the previously bound features is reactivated. The account proposes that complete stimulus-response (S-R) repetitions improve performance whereas partial repetitions (i.e., a response repetition in absence of a stimulus repetition) impair performance because previously bound features interfere with current stimulus features, thereby providing an alternative explanation of the Gratton effect (35). The flanker task comprises complete and partial S-R repetitions. While 50% of cC and iI trials contain exact S-R repetitions, none of iC and cI trials include exact response repetitions (36). Partial repetitions are present in 50% of iC and cI trials but completely absent in cC and iI trials (37). Several authors argue that conflict adaptation is partially confounded by performance benefits obtained by exact S-R repetitions (response repetition trials) (36). For this reason, it is necessary to analyze trials without response repetitions (response change trials) to obtain effects of conflict adaptation (38). Nonetheless, conflict adaptation has been demonstrated in the absence of S-R repetitions suggesting that repetition effects cannot fully account for the Gratton effect (39, 40). For simplicity, conflict adaptation in response change trials will be referred to as “pure” conflict adaptation in the following as it is not confounded by performance benefits obtained by exact S-R repetitions (36, 37). Conflict adaptation in response repetition trials will be referred to as “impure” conflict adaptation. While conflict adaptation is a common measure in cognitive control tasks (41), it has barely been studied in ASD. One study found conflict adaptation in adolescent with high-functioning ASD but studies on adults with ASD are missing (42).

Another key component of cognitive control is the ability to adapt behavior after an error by monitoring performance and selecting contextually appropriate responses while simultaneously inhibiting inadequate response tendencies (20). Errors can be understood as unintentional deviations from routine behavior after a long sequence of correct responses in the flanker task. The ability to monitor and respond to errors can be quantified by post-error slowing (PES), which refers to the relative slowing of RT in trials that follow an error compared to correct trials (43, 44). Previous studies demonstrated reduced PES in individuals with ASD, shown for children (45, 46) and adults (47), compared to HC. On the contrary, Sokhadze et al. (46) found similar PES across these groups. Several authors propose that individuals with ASD face difficulties detecting errors and struggle to implement compensatory strategies (28, 30) or adjust response strategies after an error (22, 45). Prior studies suggest that individuals with ASD have difficulties to monitor behavior consistently and accurately (17, 30, 48). These deficits may be associated with difficulties to disengage form a task or situation and adapt behavior to new requirement (25) and possibly contribute to problems in directing behavior toward a desired outcome (30, 45, 49). These deficits in error processing may be explained by an insensitivity to response outcomes and have been linked to restricted and repetitive behavior in ASD (22, 45, 47).

To gain a better understanding regarding the underlying cognitive processes and cognitive changes of error processing in ASD, the study of electrophysiological correlates is necessary. The anterior cingulate cortex (ACC) is crucial to cognitive control processes as it detects conflicts, initiates the upregulation of cognitive control, and thereby enables improved task performance (21). Besides, the ACC is of importance regarding self-monitoring abilities (48) and strongly associated with error awareness, which is essential to adapt behavior after an error (50). Deficits in ACC function relate to symptoms of ASD such as difficulties in the regulation of attention and rigidity in behavior as well as social–emotional and social-cognitive deficits (32, 45, 48, 49). Alongside the ACC, the anterior insula (AI) is relevant to cognitive control processes because its activity reflects a reaction to salient events (e.g., errors) and allows for the subsequent initiation of appropriate control signals (50–52). In individuals with ASD, abnormalities in AI structure and function have been reported (53–55). Given these findings, shedding light on ACC and AI function is necessary to provide a comprehensive insight of cognitive control in ASD. For this purpose, error-related potentials (ERPs) such as error-related negativity (ERN) and error positivity (Pe) (56, 57) are examined. In line with the CMT, recent research argues that the ERN reflects conflict detection. Changes in the ERN amplitude possibly represent dynamic changes in response conflict that depend on the degree of competition between correct and incorrect response options. Enhanced ERN amplitudes presumably occur in case of high response conflict (58). The Pe reflects error awareness, i.e., the conscious recognition of an error and the subsequent adaptation of corrective cognitive and behavioral processes (56). Most studies report reduced ERN amplitudes in children and adolescents (22, 42, 45, 46, 59) as well as adults (32) with ASD compared to HC. Sokhadze et al. (59) suggest that the altered functional connectivity in ASD may explain why individuals with ASD process information focusing on details rather than on the global level (60). In the context of cognitive paradigms, the authors assume that this perceptual style may lead to more effortful processing of individual trials. Consequently, after an error, fewer cognitive resources may be available to process the error adaptively and initiate behavioral adjustment (59), possibly explaining the altered ACC activity (47) that presumably contributes to difficulties in error detection and behavioral adjustment after an error (22, 28, 30, 45). Contradictory to these findings, other studies indicate no alterations of ERN amplitudes in ASD (61–63) and two studies found increased ERN amplitudes in children with ASD in comparison to HC. In this regard, it is important to highlight that increased ERN amplitudes were only reported in children with ASD that demonstrated a verbal IQ equal to or greater than 103 (30), or an IQ of 85 or higher (64) implying that error-related brain activity may vary depending on IQ. These findings suggest that there may be differences in individuals with high-functioning autism compared to other individuals on the autism spectrum with regard to error processing. Research on Pe amplitudes in ASD is even more inconclusive. Two studies reported significant decreases in Pe amplitudes in children (45) and adults with ASD (32) compared to HC. In contrast, other authors found similar Pe amplitudes in individuals with ASD and HC (22, 46, 59, 61). Two recent meta-analyses conclude that most evidence points toward reduced ERN amplitudes in individuals with ASD. Pe amplitudes in individuals with ASD require further research due to the small number of studies conducted to date. This shortcoming is above all evident in studies examining ASD in adults, as only one study on adults with ASD could be included in the meta-analyses (29, 65). Currently, it is uncertain whether the alterations of error processing reported in children and adolescents with ASD persist into adulthood. On the behavioral level, initial evidence suggests that PES increases with age in HC (66). Whether individuals with ASD show a similar development is unknown. McMahon and Henderson (28) investigated how error-processing abilities change from childhood to late adolescence in social and nonsocial contexts. They found larger ERN amplitudes in older participants compared to younger participants in HC and individuals with ASD. Thus, they hypothesized that error-monitoring abilities improve with age, but, regardless of age, ERN amplitudes were still smaller in the ASD group in the nonsocial task. Owing to the lack of studies conducted on adults with ASD, it is uncertain how ERN and Pe evolve in adulthood (29, 65). First studies suggest a relationship between error-related brain activity and general cognitive ability (measured as IQ) in children with ASD (30, 64). However, no study to date investigated whether the described alterations also exist in adults with ASD that are not affected by delays of cognitive development as defined in the ICD-10 diagnostic classification (67).

We examined cognitive control and error processing in adults with the aforementioned subtype of ASD to assess whether the evidence pointing toward altered error processing in children and adolescents with ASD manifests in this subpopulation of ASD patients. We focused on the generation of routine behavior in the presence of response alternatives and investigated how behavior and electrophysiological activity change subsequent to either solved conflicts or errors which equate unintentional deviations from routine. To achieve this, we conducted a modified version of the Eriksen flanker task. Since previous studies have shown that individuals with ASD show congruency effects in the Flanker Task and there is preliminary evidence indicating that conflict adaptation is present in adolescents with high-functioning ASD, we hypothesized that adults with ASD would show comparable congruency effects and conflict adaptation compared to HC. In accordance with previous studies, we further assumed that individuals with ASD would show an altered reaction to errors, indicated by decreased PES, in contrast to HC. Because previous research points toward changes in electrophysiological processing of errors in ASD, we expected reduced ERN and Pe amplitudes in the ASD group compared to HC.

## 2. Methods

### 2.1. Participants

We examined *N* = 17 healthy controls (HC) and *N* = 17 adults with a subtype of ASD without any delay in language processing or cognitive development, formally referred to as Asperger’s syndrome (AS) (67, 68). Individuals with ASD were recruited during outpatient consultations at Hannover Medical School. ICD-10 criteria of AS (67) were thoroughly explored by a self-developed semi-structured interview (“Diagnostic interview: AS in adulthood”). After a general section focusing on medical anamnesis (somatic, psychiatric, and social histories, including childhood development), the interview continued with a special section involving AS that included the following items with respect to childhood and adulthood: social interaction and communication (e.g., friendships with/relationship to/interest in peers, and being a loner and suffering from loneliness); special interests (e.g., spending leisure time and interest in specific objects/topics); stereotypic behavior (e.g., rituals and reaction toward disturbances of rituals); and other characteristics (e.g., clumsiness and sensitivity toward noises/smells/tactile stimuli). The interview contained items and descriptions of all relevant criteria for the diagnosis of AS as defined in ICD-10 (67). The result of the interview was confirmed for every AS-subject by verifying the threshold value of the German version of the autism-spectrum quotient (69) and the empathy-quotient (70). Additionally, we observed eye contact, facial expression, prosody, and “mirroring” of affections and clumsiness during the interview. The interview was conducted by the same experienced psychiatrist and had a duration of approximately 90 min. At the time of diagnostic investigation, the investigator was blind to the research questions. Diagnosis was completed with information from personal interviews, gained by telephone or in written form, of observers in child- and/or adulthood (e.g., partners, friends, and parents or siblings). In some cases, school reports were incorporated. All ICD-10 criteria had to be clearly fulfilled to confirm diagnosis (67).

All patients lived independently; some lived alone and had a regular profession. All of them had a mild form of autism corresponding to the level 1 of DSM-V classification (1). None of the ASD participants had comorbid ADHD. There were no other exclusion-criteria based on psychiatric comorbidities or medication.

HC were recruited *via* notice boards at Hannover Medical School and a newspaper advertisement. All HC underwent the same diagnostic assessment as ASD participants. None of the HC showed signs of AS or had a medical history of neurological or psychiatric disease.

All participants were native German speakers and reported normal or corrected-to-normal vision. The groups of participants were matched for age, gender, and verbal IQ that was assessed using the German version of the multiple-choice vocabulary test (MWT-B) (71). To ensure that there were no significant differences between the groups, statistical tests were performed, the results of which and sample characteristics are shown in Table 1.

**Table 1:** Sample characteristics for healthy controls (HC) and participants with autism spectrum disorder (ASD).

	HC		ASD		Significance
**Characteristic**	**M**	**SD**	**M**	**SD**	
N (male, female)	17 (9, 8)		17 (9, 8)		n.s.
Age in years	39.8	12.8	39.8	8.6	*t*(32) = −0.01, n.s. (*p* = 0.99)
Verbal IQ	106	3.1	109	7.7	*MWU* = 188.5, n.s. (*p* = 0.13)
Autism Quotient (AQ)			37.6	6.9	
Empathy Quotient (EQ)			17.1	7.3	

The study was conducted at Hannover Medical School and approved by the Ethics Committees of Hannover Medical School and the University of Lübeck. All participants gave written informed consent in accordance with the Declaration of Helsinki and received a monetary reimbursement.

### 2.2. Stimuli and paradigm

The participants completed a modified version of the Eriksen flanker Task (31). An array of seven white capital letters (“courier new” font) was displayed against a dark background. Flanker stimuli covered 2.1 °of visual angle in width and were presented on a 17’-CRT-monitor on a Windows computer using Presentation software (Neurobehavioral Systems, Inc., Albany, CA). The participants were instructed to respond as fast and accurately as possible to the target letter in the middle with their left or right index finger. Each block comprised equal amounts of congruent (HHHHHHH, SSSSSSS) and incongruent (SSSHSSS, HHHSHHH) trials in random order.

**Figure 1:**
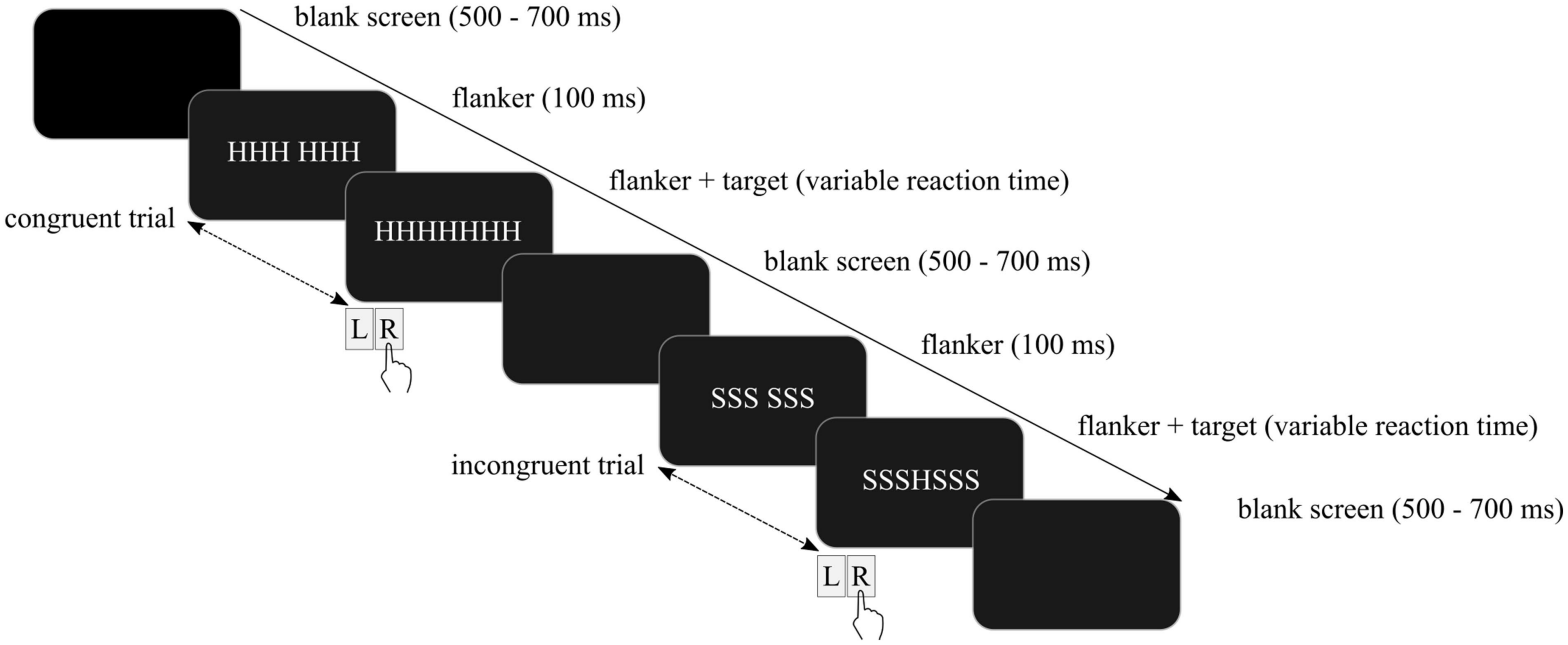
Schematic depiction of the modified Eriksen flanker task including a congruent trial followed by an incongruent trial.

All participants completed a practice session of 30 trials including feedback about the accuracy of the given response. The practice session was repeated when errors occurred in more than 20% of trials. Upon completion of the practice session, the experiment started automatically. All trials began with a dark empty screen. Flanker stimuli were presented after 500–700 ms depending on the trial type. Similarly to previous studies, target stimuli were presented with a delay of 100 ms to maximize facilitating and interfering effects of flanker stimuli (42, 72). The trial was completed as soon as a response was provided, and the next trial started. Contrary to the practice session, feedback regarding the response accuracy was not provided. The response–stimulus-interval varied randomly between 600 ms and 800 ms. Examples of two trials are depicted in Figure 1.

The experiment included a total of 1,010 trials divided into 10 blocks comprising 101 trials each. Feedback on the mean reaction times was given after every block. The participants were instructed to respond as fast as possible after the first block. In every other block, the instruction varied depending on reaction times (RT) in the current block compared with the preceding block. If participants slowed down, they were notified about their slowing in RT and asked to respond slightly faster. If responses accelerated, the participants were informed that their RT were appropriate and instructed to keep responding fast and accurately. This feedback aimed to ensure fast responses.

### 2.3. EEG acquisition

The experiment was performed in a separate EEG recording chamber with dimmed light. An ActiveTwo head cap and the ActiveTwo BioSemisystem (BioSemi B. V., Amsterdam, Netherlands) were used to capture EEG activity. Signals were recorded from 32 electrodes according to the 10/10 system, utilizing active electrodes in an elastic cap. EEG signals were recorded with a sampling rate of 512 Hz and stored utilizing the corresponding ActiView software package (BioSemi). Data were high-pass filtered at 0.1 Hz and a notch filter peaking at the online frequency of 50 Hz was applied. Blink artifacts were corrected using an internal model of eye artifact topographies as implemented in BESA software using the virtually created vertical and horizontal electrooculogram channels. Two electrodes were added and used as reference and ground electrodes during recording which is common for BIOSEMI (common mode sense, CMS, and driven right leg, DRL). The blind component separation, SOBI, was used to correct for artifacts caused by eye movements and blinks (73) as it performed more accurately compared with other artifact corrections (74). The method is based on blind source separation for the removal of ocular artifacts from EEG data which comprises automated independent component analysis. All remaining artifacts were removed by visual inspection of individualized peak-to-peak amplitude criteria. ERPs were Iow-pass filtered at 30 Hz to remove high-frequency noise. Separate grand-average ERPs were generated for both groups of participants. A baseline of 200 ms before target onset and 100 ms prior to the presentation of flanker stimuli was chosen for response-locked ERPs. To ensure that sequential effects in correct trials were not disturbed by cognitive processes caused by errors or processes not related to sequence processing, only sequences starting 3 s after an error were included in the analysis. In addition, impulsive (RT < 200 ms) and delayed responses were excluded from analysis. EEG data were preprocessed using current versions of EEGLab (75) and the EEGLab plugin ERPlab (76).

The error-related negativity (ERN) and the error positivity (Pe) were measured as the difference between correct and incorrect trials. This procedure was recommended in a recent meta-analysis (29). Consistent with previous research, analyses were conducted over fronto-central and centro-parietal electrodes (Fz, Cz, Pz) (56, 57). The ERN was measured as the most negative peak in the time window of 0 to 100 ms and the Pe as the peak maximum amplitude between 200 and 500 ms (56).

### 2.4. Data analysis

All statistical analyses were performed using R (77) and all figures were plotted with ggplot2 (78).

A Mann–Whitney U test was performed to test differences in accuracy between individuals with ASD and HC. The planned t-test for independent samples could not be conducted, because the assumption of normal distribution of the residuals was not met (Shapiro–Wilk test: *p* = 0.001). Group differences in congruency effects were examined with a 2 × 2 × 2 mixed analysis of variance (ANOVA) on mean RT with the between-subject factor group (ASD, HC) and the within-subject factors congruency (congruent, incongruent) and response type (correct, error). The size of conflict adaptation was calculated separately for both groups and trials with a response change (RC) and trials with a response repetition (RR) using the following function: $ConflictAdaptation=\left(RT_{cI}-RT_{cC}\right)-\left(RT_{iI}-RT_{iC}\right).$ Positive values of conflict adaptation indicate enhanced recruitment of cognitive control processes whereas values not significantly different from zero indicate no upregulation of cognitive control (42). A 2 × 2 × 2 mixed ANOVA with the between-subject factor group (ASD, HC) and the within-subject factors prime congruency (congruent, incongruent) and probe congruency (congruent, incongruent) on mean RT in response chance trials was performed to assess “pure” conflict adaptation. Response repetition trials were not included in the ANOVA to prevent confounding caused by performance benefits obtained by exact S-R repetitions (36). Post-error slowing (PES) was measured utilizing the following function: $PES={\bar{RT}}_{post-error}-{\bar{RT}}_{post-correct}$ as this method is the most popular one (79) and thereby ensures comparability with prior studies. A Mann–Whitney U test was conducted to assess differences in PES between individuals with ASD and HC. A t-test could not be performed because the residuals were not distributed equally as indicated by a significant Shapiro–Wilk test (*p* = 0.001). ERN and the Pe difference amplitudes were analyzed with separate 2 × 3 × 2 mixed ANOVAs with the between-subject factor group (ASD, HC) and the within-subject factors electrode (Fz, Cz, Pz) and probe congruency (congruent, incongruent). Partial eta squared (*η_p_*^2^) was reported as an effect size. Whenever appropriate, Bonferroni-adjusted pairwise comparisons were performed subsequently to significant ANOVA results.

## 3. Results

### 3.1. Behavioral results

#### 3.1.1. Congruency effect

**Figure 2:**
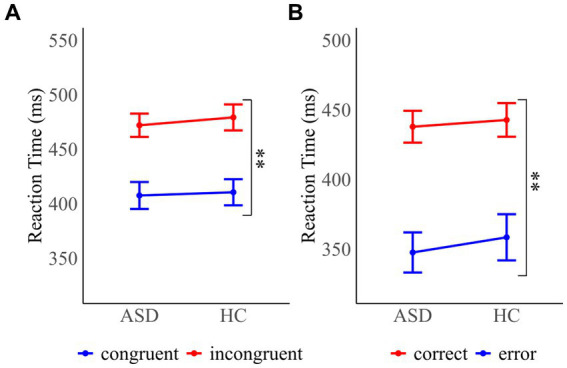
Mean reaction times for **(A)** congruent and incongruent probe trials for participants with autism spectrum disorder (ASD) and healthy controls (HC) **(B)** correct and error responses in ASD and HC participants. Error bars reflect the standard error. *p < 0.05 and **p < 0.01.

Mean reaction times (RT) for congruent and incongruent trials as a function of group are depicted in Figure 2A. The congruency effect was examined using a 2 × 2 mixed ANOVA with the between-subject factor “group” and the within-subject factor “congruency” on mean RT. It revealed a significant main effect of congruency (*F*(1, 32) = 393.60, *p* < 0.001, *η_p_*^2^ = 0.92), confirming significantly faster RT in congruent (*ASD* (*M* ± *SD*): 408 ± 51 ms; *HC*: 411 ± 49 ms) compared with incongruent trials (*ASD*: 472 ± 44 ms; *HC*: 479 ± 49 ms). The main effect of group (*F*(1, 32) = 0.10, *p* = 0.76) and the interaction of group and congruency (*F*(1, 32) = 0.40, *p* = 0.53) were not significant, demonstrating that adults with autism spectrum disorder (ASD) and healthy controls (HC) showed the expected congruency effect.

#### 3.1.2. Accuracy

Mean RT as a function of accuracy are shown in Figure 2B. A Mann–Whitney U test was performed to assess differences in error rates between individuals with ASD and HC. The test was nonsignificant (*MWU* = 115, *p* = 0.318), confirming that errors were equally frequent across groups (*ASD*: 5.3 ± 4.3%; *HC*: 6.2 ± 4.0%). A 2 × 2 mixed ANOVA on mean RT with the between-subject factors “group” and the within-subject factor “response accuracy” (*F*(1, 32) = 192.98, *p* < 0.001, *η_p_*^2^ = 0.86) showed that RT were significantly faster in error trials (*ASD*: 348 ± 59 ms; *HC*: 359 ± 68 ms) in comparison to correct trials (*ASD*: *438* ± 47 ms; *HC*: 443 ± 50 ms). RT were comparable across groups as demonstrated by a nonsignificant main effect of group (*F*(1, 32) = 0.18, *p* = 0.672) and interaction of group and trial type (*F*(1, 32) = 0.23, *p* = 0.636).

#### 3.1.3. Conflict adaptation

**Figure 3:**
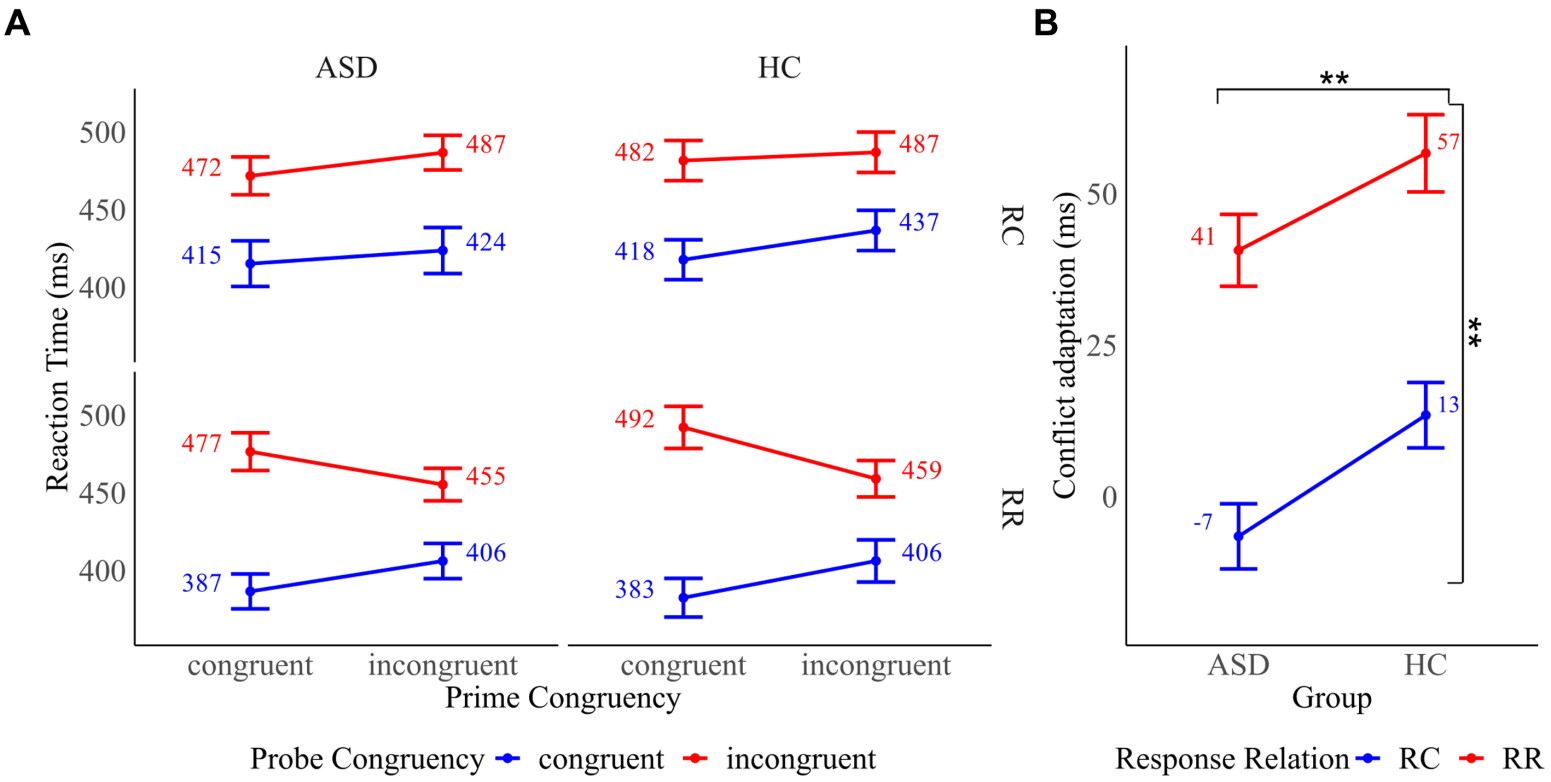
**(A)** Mean reaction times for all prime and probe trial combinations by response relation (response change (RC) and response repetition (RR)) in participants with autism spectrum disorder (ASD) and healthy controls (HC). **(B)** Conflict adaptation in RC and RR trials in participants with ASD and HC. Error bars reflect the standard error. *p < 0.05 and **p < 0.01.

Mean RT of all prime and probe trial combinations and mean conflict adaptation effects as a function of group are presented in Figure 3. To assess “pure” conflict adaptation, we conducted a 2 × 2 × 2 mixed ANOVA with the between-subject factor “group” and the within-subject factors “prime congruency” and “probe congruency” on RT in response chance trials. The analysis revealed a significant main effect of prime congruency (*F*(1, 32) = 29.05, *p* < 0.001, *η_p_*^2^ = 0.48) and probe congruency (*F*(1, 32) = 256.49, *p* < 0.001, *η_p_*^2^ = 0.89), indicating that RT were faster in congruent compared to incongruent trials. The main effect of the group was not significant (*F*(1, 32) = 0.13, *p* = 0.725). The group x prime congruency interaction (*F*(1, 32) = 0.01, *p* = 0.932), the group x probe congruency interaction (*F*(1, 32) = 0.14, *p* = 0.713) and the prime congruency x probe congruency interaction (*F*(1, 32) = 0.84, *p* = 0.366) did not reach significance. There was a significant three-way interaction of group, prime congruency and probe congruency (*F*(1, 32) = 6.88, *p* = 0.013, *η_p_*^2^ = 0.18). Decomposition of the interaction revealed that conflict adaptation was present in HC as shown by a significant interaction of prime and probe congruency (*F*(1, 16) = 6.25, *p =* 0.024, *η_p_*^2^ = 0.28) in this group. In contrast, the interaction of prime and probe congruency was not significant in the ASD group (*F*(1, 16) = 1.46, *p =* 0.245), implying that conflict adaptation was absent in these participants.

#### 3.1.4. Post-error slowing

**Figure 4:**
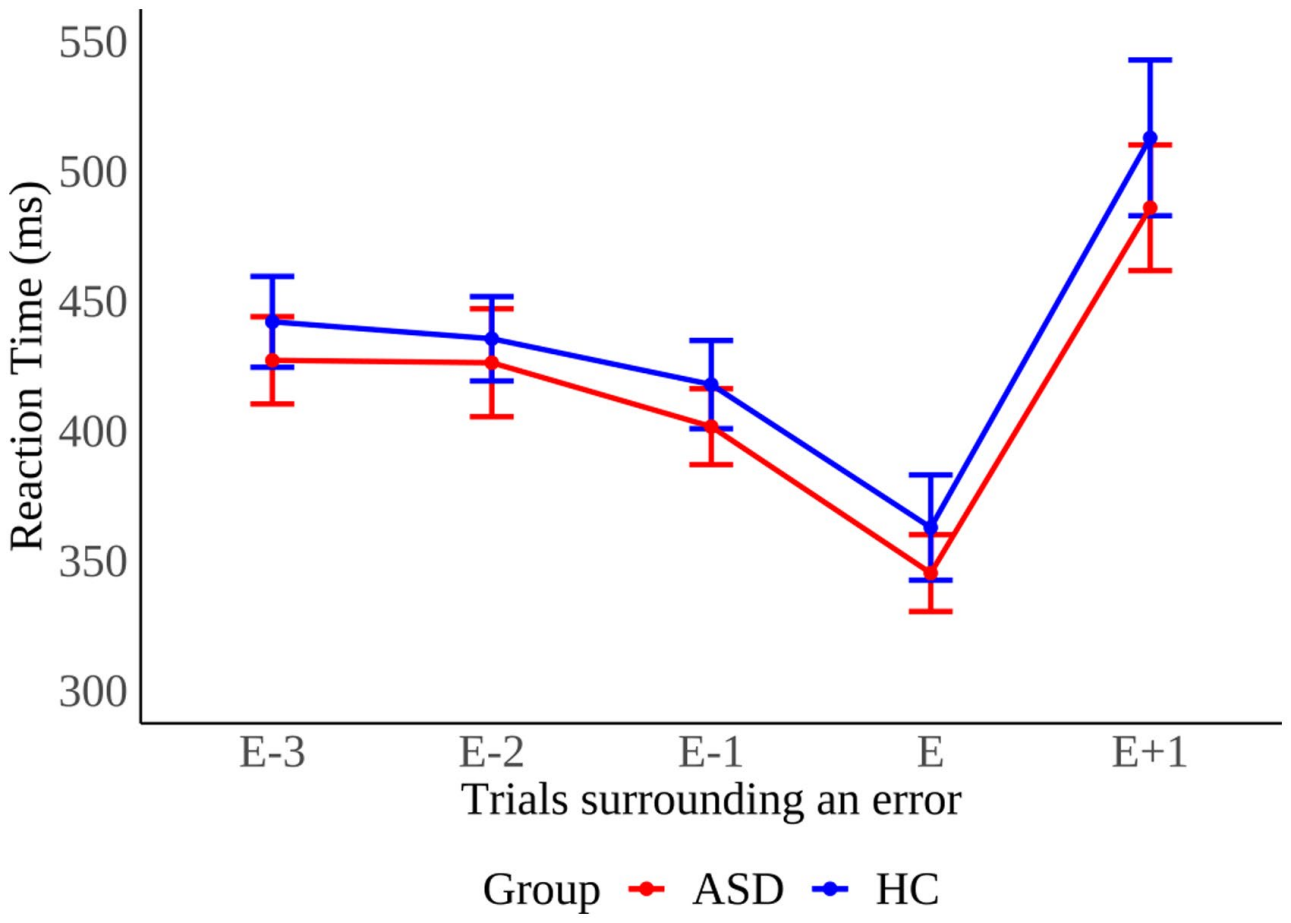
Pattern of mean reaction times in trials before (E-n), during (E), and after (E+1) error commission in individuals with autism spectrum disorder (ASD) and healthy controls (HC). Error bars reflect the standard error.

The pattern of mean RT in trials before and after error commission is shown as a function of group in Figure 4. We calculated post-error slowing (PES) by subtracting RT of post-correct trials from RT of post-error trials as described in section “Data analysis”. We performed a between groups Mann–Whitney U test to examine whether PES differed across groups. The test did not reach significance (*MWU* = 121, *p* = 0.433), implying that PES was comparable across groups (*ASD*: 45 ± 78 ms; *HC*: 60 ± 80 ms).

### 3.2. Electrophysiological results

**Figure 5:**
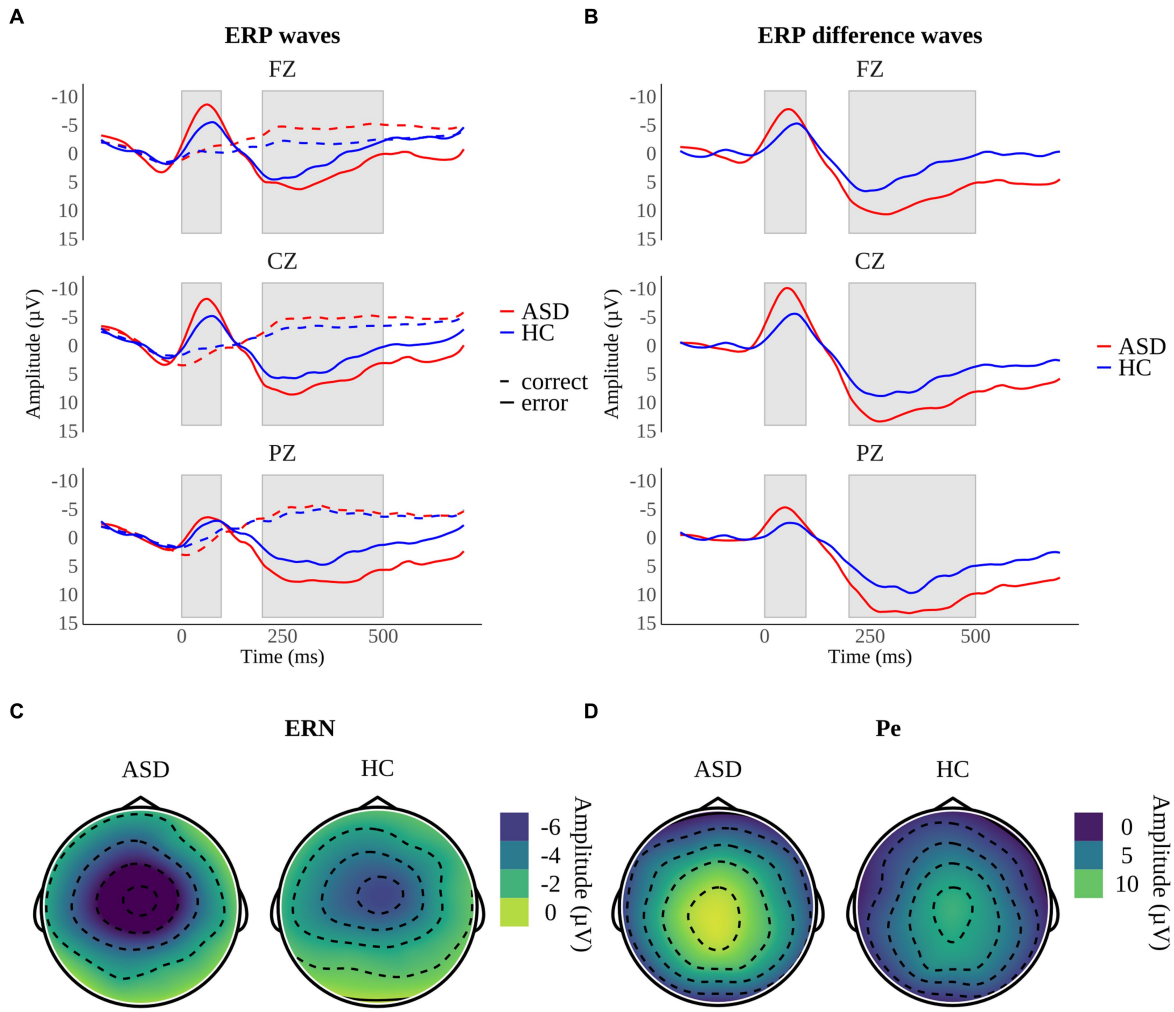
Response-locked grand average ERPs on midline electrodes for **(A)** correct responses and error responses and **(B)** difference waveforms (error minus correct responses) in participants with autism spectrum disorder (ASD) and healthy controls (HC). Shaded areas reflect the time interval of the error-related negativity (ERN) between 0 and 100 ms and the error positivity (Pe) between 200 and 500 ms. Topographical maps depict the mean amplitudes of **(C)** the ERN at 56–64 ms and **(D)** the Pe at 248–300 ms.

Response-locked grand average ERP waveforms as well as topographical maps of midline electrodes for ASD and HC participants are displayed in Figure 5.

#### 3.2.1. Error-related negativity

We performed a 2 × 2 × 3 mixed ANOVA with the between-subject factor “group” and the within-subject factors “congruency” and “electrode” to examine ERN difference amplitudes. The ANOVA demonstrated a significant main effect of electrode (*F*(2, 64) = 31.74, *p* < 0.001, *η_p_*^2^ = 0.50). Bonferroni-adjusted pairwise comparisons revealed a smaller ERN peak minimum difference amplitude of electrode Pz compared with Cz (*p* < 0.001) and a trend toward a larger peak minimum difference amplitude on electrode Fz compared with Pz (*p* = 0.05). Amplitude differences between Cz and Fz were not significant (*p* = 0.42). Thus, as expected, ERN difference amplitudes were larger at fronto-central electrodes compared with the parietal electrode. Main effects of group (*F*(1, 32) = 1.37, *p* = 0.251) and congruency (*F*(1, 32) = 0.01, *p* = 0.905) did not reach significance. However, we found a significant group x congruency interaction (*F*(1, 32) = 4.84, *p* = 0.035, *η_p_*^2^ = 0.13). The interaction was decomposed into main effects. The Bonferroni-adjusted main effect of group was significant in incongruent trials (*F*(1, 100) = 17.0, *p* < 0.001, *η_p_*^2^ = 0.15), implying significantly larger ERN difference amplitudes in individuals with ASD in contrast to HC in the presence of high conflict (*ASD*: −9.3 ± 6.1 μV; *HC*: −5.1 ± 3.9 μV). On the contrary, the Bonferroni-adjusted main effect of group did not reach significance in congruent trials (*F*(1, 100) = 0.65, *p* = 0.0842) indicating that ERN difference amplitudes were comparable in this condition (*ASD*: −6.5 ± 6.8 μV; *HC*: −7.5 ± 5.7 μV). No other interactions reached significance, including a group x electrode (*F*(2, 64) = 2.34, *p* = 0.104), an electrode x congruency (*F*(1.5, 48) = 2.97, *p* = 0.074) and a group x electrode x congruency interaction (*F*(1.5, 48) = 0.22, *p* = 0.743). Degrees of freedom were Greenhouse–Geisser-adjusted when necessary (Mauchly’s-Test: *p* < 0.001).

#### 3.2.2. Error positivity

Pe difference amplitudes were analyzed using a 2 × 2 × 3 mixed ANOVA with the between-subject factor “group” and the within-subject factors “congruency” and “electrode.” The main effect of electrode was significant (*F*(1.48, 47.36) = 4.09, *p* = 0.034, *η_p_*^2^ = 0.11). Peak maximum difference amplitudes were compared using Bonferroni-adjusted pairwise comparisons that indicated comparable amplitudes across electrodes (all *p* > 0.05). In addition, the main effect of group reached significance (*F*(1, 32) = 5.60, *p* = 0.024, *η_p_*^2^ = 0.15), implying that individuals with ASD showed larger Pe maximum difference amplitudes in contrast to HC (*ASD*: 10.7 ± 11.1 μV; *HC*: 5.2 ± 6.2 μV).There was no main effect of congruency (*F*(1, 32) = 0.09, *p* = 0.761). None of the interactions were significant, comprising a group x electrode (*F*(1.48, 47.36) = 1.41, *p* = 0.252), a group x congruency (*F*(1, 32) = 0.59, *p* = 0.446), an electrode x congruency (*F*(1.32, 42.24) = 0.89, *p* = 0.379) and a group x electrode x congruency interaction (*F*(1.32, 42.24) = 1.11, *p* = 0.316). Degrees of freedom were Greenhouse–Geisser-adjusted whenever appropriate (Mauchly’s-Test: *p* < 0.001).

## 4. Discussion

We found similar performance in the flanker task and congruency effects in both groups of participants, indicating that irrelevant stimuli affect the cognitive system of individuals with autism spectrum disorder (ASD) and healthy controls (HC) in a similar manner. Additionally, both groups demonstrated comparable post-error slowing (PES), suggesting that individuals with ASD and HC adjust behavior after an error was committed. In contrast to our initial assumptions, our data indicate that adults with ASD struggle to enhance cognitive control in response to a conflict as “pure” conflict adaptation was absent in these participants. Finally, our results imply that error-related electrophysiological processing is altered in adults with ASD because error-related negativity (ERN) and error positivity (Pe) difference amplitudes were enhanced in this group.

Our reports of comparable congruency effects across groups comply with the findings of several earlier studies (22, 30, 32, 63). Both groups of participants exhibited the pattern of reaction times (RT) expected according to the Conflict Monitoring Theory (CMT) (21) and Consistent with the CMT (21), HC demonstrated “pure” conflict adaption, indicating that conflict causes an upregulation of cognitive control in this group of participants. In our study “pure” conflict adaptation was absent in ASD participants, contradicting earlier findings in children and adolescents with ASD (42). In line with the CMT, our findings suggest that adults with ASD struggle to enhance cognitive control in response to a conflict (21). However, it is important to bear in mind that the absence of conflict adaptation in the ASD group does not reflect less adaptive behavior in this version of the flanker task. The probability of being confronted with a high conflict trial following a congruent trial was the same as being confronted with a low conflict trial. Therefore, the degree of conflict in the prime trial did not allow for a reasonable prediction of future events. Thus, one could argue that adjusting cognitive control in response to the degree of conflict in the previous trial does not represent the more rational response strategy in this context. In line with this, both groups of participants showed similar accuracy rates, implying that conflict adaptation was not crucial to adjust behavior and improve subsequent performance. Our results suggest that prior experiences have a reduced impact on current behavior in ASD which may contribute to less flexible behavior.

The finding of similar PES across groups challenges previous studies reporting reduced PES in children and adolescents with ASD (22, 45, 46). In contrast to previous reports, we could not find evidence for the assumption that individuals with ASD struggle to detect errors (28, 30) or subsequently adjust response strategies after an error (22, 45). An explanation for the discrepancies is that we examined a high-functioning subpopulation of ASD patients. In this population, in contrast to other forms of autism, a previous study suggests increased performance monitoring (62) which may enable adaptive adjustments of behavior in response to errors. Differences in age provide another possibility because we examined adults exclusively. In line with our results, it is plausible that in accordance with HC (66), PES increases in individuals with ASD with advancing age. However, one study conducted on adults with ASD also reported reduced PES (47). Thus, further research is needed to provide definite conclusions.

Both groups of participants showed the ERN reflected by the expected negative deflections following error trials (Figure 5B). ERN difference amplitudes were larger at fronto-central electrodes (Cz, Fz) in comparison to the parietal electrode (Pz) which is consistent with original reports on the ERN (56, 57) and the assumption that the ERN reflects activity in the anterior cingulate cortex (ACC) (80). Participants with ASD displayed larger ERN difference amplitudes indicating that conflict detection is elevated in adults with high-functioning ASD in incongruent trials. This is consistent with the assumption that changes in ERN amplitude depend on changes in response conflict caused by varying degrees of competition between correct and incorrect response tendencies (58). These findings contradict prior research reporting reduced ERN amplitudes in ASD (22, 32, 45, 46) or comparable ERN amplitudes across groups (61, 62). Two previous studies found enhanced ERN amplitudes in children with ASD that either had higher verbal abilities – i.e., a verbal IQ equal to or greater than 103 compared to children with a verbal IQ of less than 103 (30) or an IQ of 85 or higher (64). Hence, we assume our findings are explained by the fact that we only examined individuals with ASD that show a regular development of language and cognition (68). Henderson and colleagues (30) suspect that increased ERN activity reflects adaptive or compensatory processes enabling children with ASD to quickly monitor their actions and allow for more adaptive goal-directed behavior in tasks with a pre-specified goal. This interpretation fits with our results as participants with ASD and HC demonstrated comparable accuracy rates. In compliance with the CMT (21), our findings indicate the demand for conflict detection is enhanced in individuals with ASD in conflictual situations. Thus, increased ERN amplitudes possibly reflect increased performance monitoring and error processing efforts in ASD (29).

The Pe was present in both groups of participants as shown by the typical positive amplitude progression between 200 and 500 ms after an error (Figure 6B). Pe difference amplitudes were enhanced in individuals with ASD implying that these participants are more aware of having committed an error compared to HC (56). These results conflict with the findings of earlier studies that reported reduced Pe amplitudes in ASD (32, 45) or similar Pe amplitudes across groups (22, 46, 61). In one meta-analysis the authors found a relationship between error rates and Pe amplitudes. They concluded enhanced processing efforts, reflected by increased Pe amplitudes, facilitate equal accuracy across individuals with ASD and HC (29). These assumptions fit with our results as we found equal accuracy across groups. Therefore, we argue that increased Pe difference amplitudes in ASD reflect an enhanced processing effort of errors in ASD.

To date only one study investigated cognitive control in adults with ASD (32). Thus, our study provides first evidence for increased performance monitoring shown by enhanced ERN and Pe difference amplitudes in adults with ASD reflecting altered error processing in presence of high conflict. Increased error-related electrophysiological activity possibly underlies an enhanced processing effort or an extraordinary reactivity toward errors that possibly serves as a compensatory mechanism, enabling adaptive goal-directed behavior in adults with ASD.

### 4.1. Limitations and future directions

A major limitation of the described study is the rather small sample size of 17 participants per group. However, according to a recent meta-analysis, 17 participants per group are sufficient to detect a medium effect size of *f* = 0.25 with a power of 0.80 and an alpha of 0.05 when conducting a repeated-measures ANOVA with two groups and one within-subject factor (29). This complies with the design chosen in the present study. Another limitation is that our results do not generalize to the whole population of ASD patients. However, this study design allowed us to draw specific conclusions regarding cognitive control in adults with high-functioning ASD. Further research is necessary to confirm whether our findings are specific to adults with ASD or rather to the examined subpopulation of ASD patients.

Another limitation concerns the chosen experimental task. The Eriksen flanker task is commonly used to study cognitive control (31) and to elicit the ERN and the Pe (81). Nevertheless, it has been criticized when studying conflict adaptation as the findings may be confounded by low-level learning such as associative priming (36, 37). To prevent this bias, we analyzed response repetition and response change trials separately, but novel study designs may allow for a more reliable assessment of conflict adaptation. Braem et al. (41) argue that to examine cognitive control and minimize the bias of low-level learning, tasks should contain inducer items that trigger adaptive control, and separate diagnostic items that allow for the measurement of adaptive processes. Furthermore, new approaches to quantify PES possibly minimize biases due to performance fluctuations throughout the experiment or caused by influences of congruency on RT. Despite this, the traditional method of quantifying PES was chosen in the present study as it allowed for the best comparability to previous studies (79, 82). Moreover, we examined ERN and Pe as the difference between correct and incorrect trials. This procedure was selected as it complies with the approach used in the original reports of the ERN (56) and because it was recommended in a recent meta-analysis (29). However, this approach hampered the comparability to prior studies because the majority of studies included accuracy (correct vs. error trial) as a within-subject factor instead of treating the difference between correct and incorrect trials as the dependent variable (22, 28, 30, 32, 45, 46, 61).

In compliance with previous studies, we showed that individuals with ASD and HC demonstrate similar congruency effects, implying comparable adjustments in cognitive control in response to task-irrelevant stimuli. Additionally, both groups demonstrated equal PES, suggesting a regular reaction toward errors in adults with ASD. Future studies are needed to confirm whether this finding is specific to individuals with high-functioning autism and shed light on the role of age. In line with the CMT (21), the lack of conflict adaptation in adults with ASD possibly reflects difficulties to adjust cognitive control, corresponding to changing environmental demands. These alterations perhaps contribute to restricted and repetitive behavior in ASD. Future studies should focus on more recent approaches to examine adaptive control in conflict tasks, e.g., as proposed by Braem et al. (41) to allow for more reliable conclusions regarding conflict adaptation in ASD.

In contrast to the majority of earlier studies, error-related brain activity was enhanced in participants with ASD. In line with the suggestions of Hüpen et al. (29), we assume that increased ERN and Pe difference amplitudes are due to an elevated processing effort of errors in ASD. Beyond that, increased ERN amplitudes are a common finding in psychiatric disorders such as obsessive–compulsive disorder (65), anxiety disorders (83, 84), and less consistently in depression (85) and Gilles de la Tourette syndrome (65). Henderson et al. (30) reported that enhanced ERN amplitudes are linked to more self-reported internalizing problems, such as higher level of anxiety and depression, in their sample of children and adolescents with high-functioning ASD which is consistent with the assumption that increased ERN amplitudes may serve as a transdiagnostic endophenotype of internalizing disorders (86, 87). Future studies are necessary to confirm whether enhanced error-related brain activity is a common feature of adults with high-functioning autism and to clarify whether increased performance monitoring is associated with more internalizing problems in ASD. These studies should include participants from the whole autism spectrum to assess whether the findings of increased error-related brain activity generalize to the whole population of adult ASD patients or are specific to a subgroup of patients. For this purpose, additional factors such as symptom severity, intelligence, comorbidities, and medication status should be examined to allow for the identification of sample characteristics contributing to altered error processing. Furthermore, these may help clarify whether and in what way improvements in error processing relate to the reduction of ASD symptom severity with advancing age.

## 5. Conclusion

In summary, our findings imply that adults with high-functioning ASD do not enhance cognitive control in response to conflicts in the same manner as healthy participants. These alterations imply that prior experiences have a reduced impact on current behavior, which may contribute to repetitive and rigid behavior in ASD. Nevertheless, post-error adjustments of behavior appear to be intact implying that adults with ASD are capable of flexibly adjusting behavior in response to changed environmental demands. Performance monitoring was enhanced in ASD participants as shown by increased error-related brain activity, indicating an extraordinary reactivity toward errors in adults with ASD. Explaining the relation between the ability to adaptively adjust behavior after errors and increased error-related brain activity in adults with ASD, remains subject of future studies. In addition, our results demonstrate that findings on cognitive control in children and adolescents with ASD do not necessarily generalize to adults with ASD. Considering ASD is a disorder persisting throughout the entire life, our findings highlight the need for more research on adults with ASD and illustrate that it is advisable to differentiate between subpopulations of ASD patients.

## Data availability statement

The raw data supporting the conclusions of this article will be made available by the authors, without undue reservation.

## Ethics statement

The studies involving human participants were reviewed and approved by the Ethics Committee of Hannover Medical School and the Ethics Committee of the University of Lübeck. The patients/participants provided their written informed consent to participate in this study.

## Author contributions

LM participated in the processing, statistical analysis and interpretation of the data, and drafted the manuscript. AB and EG participated in the coordination of the study and drafted the manuscript. MR performed the measurement and the diagnostic investigation and sample specification. CS and GS participated in the coordination of the study and the conception of the study design. SB participated in the conception of the study and supported the data interpretation. DW conceived the study, participated in design and coordination of the study and the statistical analysis, interpreted the data, and drafted the manuscript. All authors contributed to the article and approved the submitted version.

## Funding

This work is funded by the Clinic for Psychiatry, Social Psychiatry and Psychotherapy of the Hannover Medical School and the Department of Neurology of the University of Lübeck. This publication is funded by the Deutsche Forschungsgemeinschaft (DFG) as part of the “Open Access Publikationskosten” program.

## Conflict of interest

The authors declare that the research was conducted in the absence of any commercial or financial relationships that could be construed as a potential conflict of interest.

## Publisher’s note

All claims expressed in this article are solely those of the authors and do not necessarily represent those of their affiliated organizations, or those of the publisher, the editors and the reviewers. Any product that may be evaluated in this article, or claim that may be made by its manufacturer, is not guaranteed or endorsed by the publisher.
